# A descriptive study of ten-year longitudinal changes in weight and waist circumference in the multi-ethnic rural Northern Norway. The SAMINOR Study, 2003-2014

**DOI:** 10.1371/journal.pone.0229234

**Published:** 2020-02-19

**Authors:** Bjarne K. Jacobsen, Marita Melhus, Kirsti Kvaløy, Susanna R. A. Siri, Vilde Lehne Michalsen, Ann Ragnhild Broderstad

**Affiliations:** 1 Centre for Sami Health Research, Department of Community Medicine, UiT The Arctic University of Norway, Tromsø, Norway; 2 Department of Community Medicine, UiT The Arctic University of Norway, Tromsø, Norway; 3 HUNT Research Centre, Department of Public Health and Nursing, NTNU–Norwegian University of Science and Technology, Trondheim, Norway; 4 Division of Internal Medicine, Department of Medicine, The University Hospital of North Norway, Harstad, Norway; National Institute of Health and Nutrition, National Institutes of Biomedical Innovation, Health and Nutrition, JAPAN

## Abstract

The obesity epidemic is dynamic with varying secular trends and differences between countries and ethnic groups. The objective of this study was to describe the age- and sex-specific longitudinal changes in weight and waist circumference in a rural Norwegian population with a high proportion of the indigenous Sami population. Based on two population-based surveys, SAMINOR 1 (2003–2004) and SAMINOR 2 (2012–2014), we present longitudinal changes in weight and waist circumference according to age at baseline in the SAMINOR 1 Survey and sex during the 10-year period. The analyses included 1538 men and 1958 women aged 36 to 69 at baseline (birth year 1934 to 1967). Forty-one percent of the population were Sami. Both weight and waist circumference were measured. The mean weight increased 0.8 kg (95% confidence interval: 0.5, 1.1) in men and 0.3 kg (95% confidence interval: 0, 0.5) in women. In both men and women, younger individuals gained significantly more weight during the 10-year follow-up than older participants (p < 0.001). The mean weight showed a statistically significant increase in men aged 36–54 and women aged 36–49 at baseline and was statistically significantly reduced in men and women aged 60–69. The mean waist circumference increased by 6.3 cm (95% confidence interval: 6.0, 6.6) in men and 8.4 cm (95% confidence interval 8.1, 8.8) in women. The mean waist circumference increased statistically significantly from SAMINOR 1 to SAMINOR 2 in all age groups, and there was an inverse relationship between age at baseline and change in waist circumference (p < 0.001). Waist circumference increased more than can be explained by changes in weight and age during the 10-year period. The inverse relationships between age at baseline in SAMINOR 1 and the 10-year change in weight and waist circumference were found in both Sami and non-Sami participants. The findings underline the need for prevention of obesity, particularly in younger people, as it is difficult to achieve permanent weight loss.

## Introduction

An increasing prevalence of obesity remains a major concern in most parts of the world [1,2]. Recent findings, however, suggest that the prevalence of obesity in high-income countries is not increasing as fast as previously [1,3–5].

Recent longitudinal studies of the relationships between age and weight (or body mass index (BMI)) or age and waist circumference in adults give an impression of an inverse linear relationship; i.e., younger adults gain more weight and increase their waist circumference more over time than middle-aged or older adults [4,6–18]. It is in this context relevant that studies suggest that genetic predisposition interacts with the obesogenic environment. This results in higher BMI in later birth cohorts compared to earlier cohorts less exposed to environmental obesogenic factors [19,20].

In Norway, longitudinal changes in weight and waist circumference over several decades have predominantly been examined in Tromsø, a city in Northern Norway (The Tromsø Study) [9,10] and in the more rural dominated Nord-Trøndelag County (The HUNT Study) [6].

The obesity epidemic clearly is dynamic with varying secular trends (e.g. within the same country), and differences between countries (e.g. high- or low income countries) [1,2]. A further concern is that the obesity epidemic may differ by ethnic group, also comprising indigenous people. Most indigenous people worldwide have lower socioeconomic status and poorer health, including higher prevalences of childhood and adult obesity, than the non-indigenous benchmark population [21]. It has been suggested that the detrimental metabolic impact of obesity may be less pronounced in the indigenous Inuit population than among Europeans [22]. Somewhat in contrast to this, a recent study found that the relationships between some measures of overweight and total mortality were different in two Inuit populations compared with in Danes. The relationships with cardiovascular mortality was similar, however [23].

The Sami is an indigenous people who traditionally inhabited northern parts of Norway, Sweden and Finland, and Russia’s Kola Peninsula. The largest population of Sami people live in Norway (around 40,000 individuals, although the number is uncertain due to no recent census or register information) [24]. The population-based SAMINOR 1 Survey was conducted in 2003–2004 [25]. It included more than 15,000 participants, both Sami and non-Sami individuals, aged 36–79 in rural Northern Norway, and demonstrated higher BMI and lower waist circumference in Sami than in Norwegian men, whereas Sami women had higher levels of both BMI and waist circumference compared to Norwegian women [26]. There are, however, no studies describing the longitudinal changes in obesity-related measures in Sami people including only individuals with repeated measurement.

Thus, the main aim of this study was to describe the longitudinal changes in weight and waist circumference during a 10-year period from 2003–2004 to 2012–2014 according to baseline age and sex in a rural, multiethnic population in Northern Norway. A secondary aim was to evaluate whether these changes differ in Sami and non-Sami participants living in the same geographic areas.

## Material and methods

### The SAMINOR study

The Population-based Study on Health and Living Conditions in Regions with Sami and Norwegian Populations–The SAMINOR Study is run by the Centre for Sami Health Research at UiT The Arctic University of Norway. The first survey of the SAMINOR Study (SAMINOR 1) was conducted in 2003–2004, in collaboration with the Norwegian Institute of Public Health [25]. The survey took place in 24 rural municipalities and districts in Northern and Central Norway with a known Sami population. All residents in the predefined regions aged 36–79 were invited [25].

During 2012–2014, the Centre for Sami Health Research conducted the SAMINOR 2 Clinical Survey (hereafter referred to as SAMINOR 2) in 10 of the 24 municipalities included in SAMINOR 1. The 10 rural municipalities are all situated in the three northern-most counties in Norway, north of the Arctic Circle. All residents aged 40–79 were invited [27]. The Sami are overrepresented in the SAMINOR surveys (as compared to the population in Northern Norway as a whole and to the total Norwegian population) because the municipalities were selected due to a high proportion of Sami inhabitants. The choice of municipalities was based on common knowledge about where the Sami people live and historical information from a census conducted in 1970.

### Methods

Both SAMINOR 1 and 2 included self-administered questionnaires and a brief clinical examination including blood samples. In SAMINOR 1, the clinical examinations took place on two research buses, placed at central locations in the municipalities. In SAMINOR 2, temporary examination sites were established in central venues. Depending on the size of the population, data collection in both surveys lasted 1–7 weeks in each of the 10 municipalities included in SAMINOR 2.

Height and weight were measured using an electronic height and weight measuring device (DS-102 in SAMINOR 1 and DS-103 in SAMINOR 2, Dongsahn Jenix, Seoul, Korea) with the participants wearing light clothing without shoes. Height was measured to the nearest 0.1 cm, and weight to the nearest 100 grams. Waist circumference was measured at the umbilicus to the nearest cm with the participant standing and breathing normally [25,27].

Using the unique personal identification number assigned to all residents in Norway, Statistics Norway linked the two surveys and identified those who had participated in both SAMINOR 1 and 2. The present analyses included participants with birth years from 1934 to 1967 (aged 36–69 in SAMINOR 1) who had attended both SAMINOR 1 and SAMINOR 2 and lived in the 10 municipalities included in SAMINOR 2 at both points in time. In SAMINOR 1, there were 11,173 invitees within these selected municipalities and birth cohorts, whereof 6317 persons (56.5%) attended the clinical examinations. In SAMINOR 1, women had a higher attendance rate than men and middle-aged (aged 46–65) higher than younger and older individuals [25]. In SAMINOR 2, there were 10,330 invitees in the birth cohorts 1934–1967, whereof 5191 (50.3%) attended. As found for SAMINOR 1, non-attenders to SAMINOR 2 tended to be men and relatively young [27]. A total of 3538 persons had attended clinical examinations in both surveys. Reasons for not taking part in SAMINOR 2 even if attended SAMINOR 1 included both that they were not invited (because they had died, too old to be invited or moved out of the 10 municipalities) or that they were unwilling or unable to attend (non-response).

Participants who lacked height or weight measurements or had remarks concerning the height and weight measurement (e.g. pregnancy, scoliosis, measured with shoes) in one or both surveys were excluded from the analyses. In the analyses regarding change in waist circumference, participants with remarks concerning waist circumference measurements were also excluded.

Information from SAMINOR 1 concerning marital status, level of education, income, smoking history (current daily smoker, previously, or never), the frequency of use of alcohol last year (never consumed alcohol, not during the last year, a few times during the last year, 1 time per month, 2–3 times per month, 1 time per week, 2–3 times per week, 4–7 times per week), leisure time physical activity, self-evaluated general health status, whether the respondent had some selected relevant prevalent chronic diseases (myocardial infarction, angina pectoris, diabetes) (yes, no) or treatment for high blood pressure or hypercholesterolemia (currently, previously, and never) was based on individual responses on the self-administered questionnaires [25]. Physical activity in leisure time was assessed by items from the “Saltin-Grimsby” questionnaire [28], which has four levels of activity. Participants who reported that their activity was on the lowest level, sedentary, i.e.: reading, watching TV or other sedentary activities, were considered inactive.

As detailed elsewhere [29], participants were categorized into ethnic groups based on answers to questions regarding language and ethnic background: "What language(s) do/did you, your parents and your grandparents use at home?", "What is your, your father’s and your mother’s ethnic background?", and “What do you consider yourself to be?” The response options were “Norwegian”, “Sami”, “Kven” and “Other” and multiple answers were allowed. Kvens are individuals who descend from Finnish men and women who emigrated from the northern parts of Finland and Sweden to Northern Norway during 1720 to 1890 [30].

The categorization of ethnic group (Sami and non-Sami) was based on answers in SAMINOR 1 (supplemented by information from SAMINOR 2 in 78 participants with missing ethnic information in SAMINOR 1, but with information in SAMINOR 2). Participants were defined as Sami if they 1) considered themselves to be Sami, or reported a Sami ethnic background for themselves, and in addition 2) spoke a Sami language themselves or had at least one parent or grandparent that used it at home [29]. The concordance of information concerning ethnic background (being Sami or not) in SAMINOR 1 and SAMINOR 2 was very high (Cohen's kappa coefficient = 0.86 (95% CI: 0.85, 0.88)). Only 6.6% of the participants reported different ethnic background in the two surveys.

### Study population

A total of 60 of the 6317 participants in the relevant municipalities and birth cohorts who attended SAMINOR 1 did not have a valid measurement of weight and waist circumference. Therefore, 6257 participants constituted the population that was followed-up. Of the 3538 who participated in both surveys, 25 lacked a valid measurement of weight or waist circumference in SAMINOR 1, and additional 17 lacked this information in SAMINOR 2. The longitudinal analyses for change in weight and waist circumference, comprising participants who had attended both SAMINOR 1 and SAMINOR 2, included 3496 participants, 1538 men and 1958 women.

### Statistical analyses

The main dependent variables in our analyses were changes in weight and waist circumference (i.e., the SAMINOR 1 value subtracted from the SAMINOR 2 value) without any adjustments for height, as for example as in BMI or waist/height-ratio. The reason is that indices with adjustments for height tend to increase with age because of the general age-related height loss [6,18,31]. Furthermore, the correlation coefficient between weight and BMI is high (r ≥ 0.83 in SAMINOR 1) and the correlation coefficient between waist circumference and the waist-to-height-ratio even higher (r ≥ 0.91 in SAMINOR 1). Another frequently used measure for abdominal obesity, the waist-to-hip-ratio, is also highly correlated with waist circumference (r ≥ 0.80 in SAMINOR 1).

We present the sex-specific relationships between age in SAMINOR 1 (which corresponds to birth cohort) and change in weight and waist circumference, respectively. We also assessed whether the change in waist circumference from SAMINOR 1 to SAMINOR 2 differed from that expected based on change in body weight and age between the two surveys, using the same approach as in another study [10]. In men and women separately, a linear regression of the relationship between waist circumference (the dependent variable) and body weight and age (the two independent variables) was performed based on the data from SAMINOR 1. Information about the body weight and age together explained 73% and 72% in men and women, respectively, of the variance of the waist circumference in SAMINOR 1 (R^2^_adjusted_). For the same men and women, we entered their values on age and weight in SAMINOR 2 into this regression equation and calculated their expected waist circumference. The difference between the observed and expected waist circumferences in SAMINOR 2 was computed. A positive difference reflects that the waist circumference has increased more than what the change in age and body weight during the 10-year follow-up can explain.

Because the municipalities were visited in a different order in SAMINOR 1 and SAMINOR 2, the time span between when the participants were examined differed from 8.7 to 11.2 years (mean 10.1 years). There was, however, no difference in the time span between the two surveys (p = 0.9) across age group in SAMINOR 1 (or birth cohort group). However, for reasons related to when the two surveys were conducted in the different municipalities, Sami participants had slightly (mean 56 days) longer time between attendance to SAMINOR 1 and SAMINOR 2 than other individuals did. Thus, when comparing longitudinal changes in weight and waist circumference in Sami and non-Sami participants, we focused on mean *annual* changes in weight and waist circumference. These comparisons were adjusted for age.

Statistical analyses included basic descriptive analyses (e.g. mean, standard deviation, 95% confidence intervals) (all tables), computations of Cohen's kappa coefficient (in order to study the concordance between information about Sami ethnicity in SAMINOR 1 and SAMINOR 2) and Pearson correlation coefficient (for assessing the correlation between the different measures of weight and overweight). We also used two-sample t-tests (to test the difference in weight and waist circumference between participants who attended both SAMINOR 1 and SAMINOR 2, men and women and Sami and non-Sami participants) and linear regression (to test for linear trends in change in weight and waist circumference over 5-years age-groups as well as interactions with sex or ethnic group). The statistical analyses were performed using SAS version 9.4 (SAS Institute, Cary, NC, USA). A p-value of < 0.05 was considered statistically significant.

### Ethics and data availability

The SAMINOR surveys were approved by the Regional Committee for Research Ethics, (REC) and the Norwegian Data Protection Authority approved data collection and storage of personal data. The Regional Committees for Medical and Health Research Ethics (REC reference 2016/176) and the SAMINOR Project Board also approved the present study. All participants gave written informed consent. The presented results are based on analyses of the much larger SAMINOR database and access to data is given for a restricted time period. Each project has to be authorized by the SAMINOR Project Board and data cannot be shared. Additionally, data cannot be shared publicly because it contains potentially identifying information. Data may be available after applications to the SAMINOR Project Board (www.saminor.no) and the Regional Committees for Medical and Health Research Ethics (https://helseforskning.etikkom.no).

## Results

The 3496 participants included in the longitudinal analyses represented 56% (52% of men and 59% of women) of the 6257 individuals born between 1934 and 1967 who had a valid measurement of weight and waist circumference in SAMINOR 1. Table 1 displays some unadjusted sex-specific characteristics of the group of 3496 participants who attended both SAMINOR 1 and SAMINOR 2 (included in the follow-up) compared to the 2761 men and women who attended SAMINOR 1 only (not included in the follow-up).

**Table 1:** Unadjusted characteristics of men and women born between 1934 and 1967 (aged 36–69 in SAMINOR 1) with information about weight and waist circumference in SAMINOR 1 (2003–2004) who were included and not included, respectively, in the follow up from SAMINOR 1 to SAMINOR 2 (2012–2014). Figures are means (standard deviation) or % (number of participants). The SAMINOR Study (n = 6257).

		Included in the follow-up	Not included in the follow-up	p-value
Men				
	No. of participants	1538^a^	1398^a^	
	Age (years)	52.8 (8.5)	51.6 (9.2)	< 0.001
	Married (%)	65.0 (999)	55.2 (771)	< 0.001
	Living with a partner (%)	79.5 (1063)	73.4 (788)	< 0.001
	No. of persons in the household	2.9 (1.4)	2.8 (1.5)	0.04
	Sami ethnicity (%)	41.0 (631)	32.8 (441)	< 0.001
	High income (%)^b^	39.7 (568)	33.0 (410)	< 0.001
	Years of education (years)	11.4 (3.7)	11.2 (3.6)	0.1
	Good/very good health (%)	73.6 (1120)	67.9 (938)	< 0.001
	History of myocardial infarction (%)	3.5 (51)	5.7 (76)	0.004
	History of angina pectoris (%)	4.4 (65)	8.1 (108)	< 0.001
	Diabetes (%)	3.0 (44)	4.0 (53)	0.1
	Ever use of drugs for hypertension (%)	19.8 (302)	19.7 (269)	0.9
	Ever use of drugs for high cholesterol (%)	15.7 (236)	17.4 (238)	0.2
	Inactive (%)^c^	20.1 (289)	24.3 (313)	0.009
	Smoking			
	Current (%)	28.8 (440)	39.0 (539)	< 0.001 ^d^
	Ex-smoker (%)	41.1 (629)	34.3 (474)	
	Never (%)	30.1 (460)	26.8 (370)	
	Alcohol consumption > once a month	51.8 (782)	52.4 (713)	0.8
	Weight (kg)	82.8 (12.6)	83.3 (14.2)	0.3
	Waist circumference (cm)	92.8 (9.7)	93.4 (10.9)	0.1
Women				
	No. of participants	1958^a^	1363^a^	
	Age (years)	52.1 (8.6)	51.6 (9.7)	0.1
	Married (%)	66.6 (1304)	58.8 (802)	< 0.001
	% Living with a partner (%)	79.9 (1356)	75.0 (832)	0.003
	No. of persons in the household	2.8 (1.4)	2.8 (1.4)	0.09
	Sami ethnicity (%)	40.4 (789)	29.2 (383)	< 0.001
	High income ^b^	34.5 (591)	28.7 (333)	0.001
	Years of education (years)	11.7 (4.0)	11.4 (4.1)	0.06
	Good/very good health	70.0 (1348)	63.9 (852)	< 0.001
	History of myocardial infarction (%)	0.8 (14)	1.9 (24)	0.005
	History of angina pectoris (%)	3.0 (56)	4.5 (58)	0.03
	Diabetes (%)	3.4 (64)	5.5 (71)	0.005
	Ever use of drugs for hypertension (%)	20.8 (403)	23.5 (315)	0.07
	Ever use of drugs for high cholesterol (%)	12.6 (241)	15.9 (212)	0.007
	Inactive (%)^c^	21.1 (379)	24.5 (301)	0.03
	Smoking			
	Current (%)	30.3 (589)	40.4 (546)	< 0.001 ^d^
	Ex-smoker (%)	31.5 (613)	26.5 (358)	
	Never (%)	38.2 (744)	33.0 (446)	
	Alcohol consumption > once a month	36.3 (693)	32.8 (428)	0.04
	Weight (kg)	70.7 (12.2)	71.7 (13.5)	0.02
	Waist circumference (cm)	84.7 (11.5)	85.6 (12.3)	0.03

^a^ Due to some missing values for most variables (except age, sex, weight and waist circumference), the number of participants included in most analyses is lower.

^b^ Yearly gross household income > 450 000 NOK

^c^ Typical leisure time physical activity includes only reading, watching TV and other sedentary activities. No regular physical activity (like walking) > 4 hours a week.

^d^ p-value for linear trends over the smoking categories

Participants with valid data from both SAMINOR 1 and SAMINOR 2 were slightly older and more likely to be living with a partner, being Sami, having a relatively high income and education as well as having a good physical health, being physically active in leisure time and being a non-smoker. They also had lower weight and lower waist circumference (Table 1).

When comparing age-adjusted mean weight in individuals who attended both surveys to the weight in those who attended only the first survey, the difference was -0.3 kg (p = 0.5) and -1.2 kg (p = 0.01), in women and men, respectively. In sex- and age-group specific analyses, the statistically significant differences were -3.0 kg (p = 0.02) in men aged 40–44; -2.8 kg (p = 0.04) in women aged 36–39; and -2.4 kg (p = 0.04) in women aged 50–54.

Similarly, in age-adjusted analyses the difference in waist circumference in participants attending both surveys compared to those who attended SAMINOR 1 only was -0.6 cm (p = 0.08) and -1.1 cm (p = 0.01) in men and women, respectively. In sex- and age-group specific analyses, the only statistically significant difference between consistent attenders and those who attended SAMINOR 1 only, was– 2.1 cm (p = 0.04) in men aged 40–44.

In the sample of 3496 participants who attended both SAMINOR 1 and SAMINOR 2, the mean BMI in SAMINOR 1 was 27.7 kg/m^2^ (95% CI: 27.5, 27.9) in both men and in women. The prevalences of general obesity (BMI ≥ 30.0 kg/m^2^) in SAMINOR 1 were 23.2% (95% CI: 21.1, 25.3) and 29.2% (95% CI: 27.1, 31.2) in men and women, respectively. The mean BMI in SAMINOR 2 was 28.0 kg/m^2^ (95% CI: 27.8, 28.2) in men and 27.9 kg/m^2^ (95% CI: 27.7, 28.1) in women. The prevalence of general obesity (BMI ≥ 30.0 kg/m^2^) in SAMINOR 2 was 27.6% (95% CI: 25.3, 29.8) and 29.8% (95% CI: 27.7, 31.8) in men and women, respectively.

Table 2 displays the sex-specific cross-sectional relationships between age and weight in SAMINOR 1 in participants who attended both surveys, as well as the results from the longitudinal analyses of change in weight between SAMINOR 1 and SAMINOR 2 according to sex and age in SAMINOR 1 (birth cohort). We found that in both men and women, the younger participants (aged 36–49) gained significantly more weight from SAMINOR 1 to SAMINOR 2 than the older participants (aged 60–69) did. There was a statistically significant increase in weight from SAMINOR 1 to SAMINOR 2 in men aged 36–54 and in women aged 36–49. The weight showed a statistically significant reduction in men and women aged 60–69 at baseline. There was no statistically significant difference between men and women in the strength of the linear inverse relationship (p-value for interaction = 0.6). Inverse relationships between age in SAMINOR 1 and change in weight between SAMINOR 1 and SAMINOR 2 were found both in participants with weight in SAMINOR 1 below and above median weight. The median change in body weight between SAMINOR 1 and SAMINOR 2 was related to age in SAMINOR 1 the same way as the mean change was.

**Table 2:** Mean (standard deviation, SD) body weight (kg) in SAMINOR 1 (2003–2004) and longitudinal changes in body weight (kg) (95% confidence interval) from SAMINOR 1 to SAMINOR 2 (2012–2014) in men and women born between 1934 and 1967 (aged 36–69 in SAMINOR 1) who attended both surveys. The SAMINOR Study (n = 3496).

	Birth year	Age in 2003 (years)	Number of participants	Mean body weight, kg (SD) in SAMINOR 1	Change in weight, kg (95% CI) between SAMINOR 1 and SAMINOR 2
Men					
	1964–1967	36–39	124	84.8 (12.9)	3.2 (2.1, 4.3)
	1959–1963	40–44	183	83.4 (12.4)	2.7 (1.8, 3.5)
	1954–1958	45–49	236	83.2 (12.2)	1.7 (1.0, 2.4)
	1949–1953	50–54	321	83.4 (13.1)	1.1 (0.5, 1.7)
	1944–1948	55–59	308	82.8 (12.7)	0.3 (-0.4, 1.0)
	1939–1943	60–64	222	81.9 (12.6)	- 0.9 (- 1.6, - 0.2)
	1934–1938	65–69	144	79.9 (11.6)	- 2.3 (- 3.2, - 1.4)
	All men	36–69	1538	82.8 (12.6)	0.8 (0.5, 1.1)
	p-value for linear trend			0.001	< 0.001
Women					
	1964–1967	36–39	163	67.8 (12.0)	3.2 (2.4, 4.1)
	1959–1963	40–44	270	69.5 (11.7)	2.1 (1.3, 2.8)
	1954–1958	45–49	342	70.8 (12.1)	1.1 (0.4, 1.7)
	1949–1953	50–54	399	70.2 (11.8)	0.2 (- 0.4, 0.7)
	1944–1948	55–59	364	71.9 (12.9)	- 0.3 (- 1.0, 0.3)
	1939–1943	60–64	241	72.1 (12.7)	- 1.3 (- 2.1, - 0.5)
	1934–1938	65–69	179	71.3 (11.6)	- 3.2 (- 4.1, - 2.2)
	All women	36–69	1958	70.7 (12.2)	0.3 (0, 0.5)
	p-value for linear trend			< 0.001	< 0.001

The results for waist circumference are displayed in Table 3. There was a statistically significant mean increase in waist circumference from SAMINOR 1 to SAMINOR 2 in all age groups and an inverse relationship between age group in SAMINOR 1 and change in waist circumference. The linear relationship (mean change over the 5-years groups) was stronger in women than in men (p-value for interaction = 0.01).

**Table 3:** Mean (standard deviation) waist circumference (cm) in SAMINOR 1 (2003–2004) and longitudinal changes in waist circumference (cm) (95% confidence interval) from SAMINOR 1 to SAMINOR 2 (2012–2014) in men and women born between 1934 and 1967 (aged 36–69 in SAMINOR 1) who attended both surveys. The SAMINOR Study (n = 3496).

	Birth year	Age in 2003 (years)	Number of participants	Mean waist circumference, cm (SD) in SAMINOR 1	Change in waist circumference, cm (95% CI), SAMINOR 1 to SAMINOR 2
Men					
	1964–1967	36–39	124	91.4 (9.5)	7.4 (6.2, 8.5)
	1959–1963	40–44	183	91.5 (9.2)	7.2 (6.2, 8.2)
	1954–1958	45–49	236	91.8 (9.0)	6.7 (5.9, 7.6)
	1949–1953	50–54	321	92.7 (10.1)	6.6 (5.9, 7.3)
	1944–1948	55–59	308	94.1 (10.3)	5.7 (5.0, 6.4)
	1939–1943	60–64	222	93.7 (9.5)	5.5 (4.7, 6.2)
	1934–1938	65–69	144	93.4 (9.5)	5.4 (4.3, 6.4)
	All men	36–69	1538	92.8 (9.7)	6.3 (6.0, 6.6)
	p-value for linear trend			< 0.001	< 0.001
Women					
	1964–1967	36–39	163	81.1 (11.1)	10.0 (8.9, 11.1)
	1959–1963	40–44	270	81.3 (10.2)	10.1 (9.2, 10.9)
	1954–1958	45–49	342	83.5 (11.2)	9.6 (8.8, 10.5)
	1949–1953	50–54	399	84.3 (10.8)	8.3 (7.6, 9.0)
	1944–1948	55–59	364	86.7 (12.3)	7.6 (6.7, 8.4)
	1939–1943	60–64	241	87.3 (11.5)	7.8 (6.8, 8.8)
	1934–1938	65–69	179	88.4 (10.9)	5.3 (4.2, 6.4)
	All women	36–69	1958	84.7 (11.5)	8.4 (8.1, 8.8)
	p-value for linear trend			< 0.001	< 0.001

S1 Table gives the corresponding results for the relationships between age group and BMI in SAMINOR 1 as well as change in BMI from SAMINOR 1 to SAMINOR 2 according to age group in SAMINOR 1. In both men and women, we found a clear linear inverse relationship between age group and change in BMI. There was no statistically significant difference between men and women in the strength of the relationship (p-value for interaction = 0.2).

Nearly 41% of the population had Sami ethnicity. The mean annual weight change, adjusted for age group, in Sami men compared with non-Sami was -0.08 kg (95% CI: -0.14,-0.02). The corresponding figure in women was -0.02 kg (95% CI: -0.08, 0.03). Similarly, we noted a borderline lower adjusted mean annual change in waist circumference in Sami than non-Sami men (-0.07 cm (95 CI: -0.13, 0)), but not in women (-0.05 cm (95% CI: -0.12, 0.02)).

S2–S5 Tables show the relationships between age in SAMINOR 1 (birth cohort) and change in weight and waist circumference in Sami and non-Sami participants. In both sexes and for both Sami and non-Sami participants, inverse relationships (p < 0.001 in most analyses) were found between age in SAMINOR 1 (birth cohort) and change in weight or waist circumference between SAMINOR 1 and SAMINOR 2. The linear relationships between age-group in SAMINOR 1 and change in weight and waist circumference were significantly stronger in Sami women than in non-Sami women (p = 0.01), whereas in men, no statistically significant differences were found (p ≥ 0.35).

Table 4 displays the mean annual change from SAMINOR 1 to SAMINOR 2 in weight (kg) or waist circumference (cm) per 5-year increase in age in SAMINOR 1. The table shows the sex-specific results in all participants and in strata of Sami and non-Sami participants.

**Table 4:** The association between age in SAMINOR 1^a^ and change in weight (kg) or waist circumference (cm)^b^ from SAMINOR 1 to SAMINOR 2 in participants aged 36–69 in 2003 who took part in both the SAMINOR 1 Survey (2003–2004) and SAMINOR 2 Survey (2012–2014). The SAMINOR Study (n = 3496).

	Men		
	All (n = 1538)	Sami (n = 631)	Non-Sami (n = 907)
Change in weight	- 0.09 (- 0.11, - 0.07)	- 0.09 (- 0.12, - 0.07)	- 0.09 (- 0.11, - 0.07)
Change in waist circumference	- 0.04 (- 0.06, - 0.02)	- 0.05 (- 0.08, - 0.02)	- 0.03 (- 0.06, - 0.01)
	Women		
	All (n = 1958)	Sami (n = 789)	Non-Sami (n = 1166)
Change in weight	- 0.09 (- 0.11, - 0.08)	- 0.12 (- 0.14, - 0.10)	- 0.08 (- 0.10, - 0.06)
Change in waist circumference	- 0.08 (- 0.09, - 0.06)	- 0.11 (- 0.14, - 0.08)	- 0.06 (- 0.08, - 0.03)

^a^ 5-year group

^b^ Mean annual change (95% confidence interval). Ethnic group was missing for 3 women.

The results shown in Table 4 are in accordance with the results given in S2–S5 Tables. Both in Sami and non-Sami men and women, there was a relatively marked and inverse relationship between age in SAMINOR 1 and change in weight and waist circumference from SAMINOR 1 to SAMINOR 2. The strength of the relationships were similar in Sami and non-Sami men. In women, there were some indications of a stronger relationship in Sami than non-Sami women (p-value for interaction between Sami and non-Sami was 0.01 for both weight and waist circumference). In accordance with this, the inverse relationship between age in SAMINOR 1 and change in BMI from SAMINOR 1 to SAMINOR 2 was statistically significantly stronger in Sami women than in non-Sami women (p = 0.001) (results not shown in tables).

The mean waist circumference in SAMINOR 2 was in men 5.8 cm (95% CI: 5.5, 6.0) larger than what was expected by changes in age and weight, and there was a modest, positive relationship between this excess waist circumference and age in SAMINOR 1 (mean 0.2 cm (95% CI: 0, 0.3) /5 year, p = 0.01). In women, the corresponding figure was 8.2 cm (95% CI: 8.0, 8.5), but we found no relationship between excess gain in waist circumference and age in SAMINOR 1 (p = 0.7).

## Discussion

In this 10-year longitudinal study, there were inverse relationships between age at baseline (in SAMINOR 1) and changes in weight and waist circumference between SAMINOR 1 and SAMINOR 2 (follow-up). Interestingly, even if the mean weight actually dropped significantly in elderly men and women (aged 60–69), the waist circumference increased significantly in all age groups. We found inverse relationships between age and change in weight and waist circumference in both Sami and non-Sami participants, although the strength of the relationships differ somewhat between the Sami and non-Sami women (Table 4, S3 Table and S5 Table). Our results confirm recent findings of an increase in mean waist circumference in both Sami and non-Sami participants in repeated cross-sectional analysis including participants who attended one or both surveys [32,33].

The younger participants had the largest weight gain. This corresponds well with results from other population-based longitudinal studies in Norway [4,6–9,11,12] and other recent studies [13,14,17,18], demonstrating an inverse relationship between age at baseline and change in body mass index and/or weight.

Longitudinal analyses in the Tromsø Study also found change in waist circumference to be inversely related to age at baseline and that the waist circumference increased statistically significantly from 1994 to 2008 in all age groups (25–69 years old) [10]. The Tromsø Study is based on a mainly urban population in the same region in Norway as the SAMINOR Study, which is based on a rural population. Our findings of an inverse relationship also support findings from Australia and Scotland [13,15].

The mean waist circumference increased more than what was explained by the change in weight and age alone. Thus, we confirm the results from other studies of an increase in waist circumference that exceeds what would be expected by increases in age and in weight or BMI. These studies were based on cross-sectional studies comparing secular trends in weight or BMI and waist circumference in the US (NHANES) [34–36], Mongolia [37] and Australia [38] as well as a longitudinal study conducted in the same region in Norway as the present study [10].

The body composition changes with advancing age. A higher proportion of the body is adipose tissue relatively to lean mass in the middle aged and elderly compared to the younger adult. Intra-abdominal adipose tissue increases more than peripheral fat mass with age and there is a substantial loss of both muscle mass and function (sarcopenia) [39,40]. A recent longitudinal study found that the rate of both subcutaneous and visceral adipose tissue accrual were larger than for body mass index, larger for visceral than subcutaneous adipose tissue, and that this rate was reduced with age for all three measures of body composition [41]. Thus, an increase in waist circumference as found in our study, may indicate an even larger relative increase in visceral adipose tissue, which is associated with insulin resistance and unfavorable metabolic alterations [42].

Our results reflect these changes in body composition. As waist circumference is more closely associated with the risk of obesity-related diseases [43–45], monitoring change in weight (or BMI) in the elderly may be less relevant than monitoring change in waist circumference when evaluating risk of disease [13,38,46].

We found that the associations between age at baseline (SAMINOR 1) and change in weight and waist circumference between SAMINOR 1 and SAMINOR 2 in Sami and non-Sami participants were qualitatively the same: inverse and statistically significant. One may note, however, that as the mean height in Sami participants is approximately 5.4 cm (3%) lower than in non-Sami, the metabolic impact of the same change in e.g. weight may be marginally larger in Sami than non-Sami.

Our findings are in accordance with a more general finding from other recent epidemiological studies, that there are only small or no differences between the Sami and non-Sami population in Norway regarding somatic health [47] and cardiovascular risk factors [32,33]. These favorable findings are usually explained by minor differences between the ethnic groups in socioeconomic factors (e.g., income, education), and cardiovascular risk factors and life style. The use of health care services has been found to be similar in Sami and non-Sami areas [48], facilitated by the publicly financed health care services available to all citizens in Norway, independent of ethnicity.

### Strengths and limitations

From an analytical point of view, it may be considered a limitation that ethnic background was self-reported. However, currently it is in Norway illegal to include ethnicity in any registry, and there is probably no other, or better, way to get this information other than asking the participants as they are the ones who knows their own identity. A recent (2019) retrospective bibliometric analysis found that relatively few studies describe how classification into race/ethnicity was done, but the large majority (90%) of those which reported the method, relied on self-report [49]. We combined self-identification with a language criterion in order to preserve some objectivity in the ethnic categorization. This definition of being a Sami resembles the criteria that have to be met in order to participate in the Sami Parliament elections. Reporting Sami home language or self-identification of being a Sami has been found to be rather stable over a 30-year period [50]. The reproducibility of information about a Sami ethnic background in our cohort was high as Cohen’s kappa was 0.86 when we compared information regarding ethnic background in SAMINOR 1 and SAMINOR 2. However, the validity may be lower, as it is likely that the long history of stigmatization and forced assimilation of Sami people may result in some Sami individuals categorizing themselves as non-Sami (Norwegian). Some individuals may not even know that they had a Sami-speaking grandparent or parent. We assume that this misclassification of ethnic background is non-differential, and it is therefore possible that the relatively minor differences in the change in weight and waist circumference according to age between the Sami and non-Sami women are somewhat underestimated.

In the present study, weight and waist circumference were not self-reported, but measured by trained personnel. This is a strength of the study as self-reported weight is likely to be underreported [51]. Waist circumference may be assessed by measuring e.g. the minimal waist circumference, at the level of the belly button or at the top of the iliac crest [45], and it is prone to measurement error [45,52] even if standardized as in the SAMINOR surveys. Thus, it is possible that the mean difference in waist circumference between SAMINOR 1 and SAMINOR 2 is somewhat biased, but we have no reason to believe that this possible bias depends on age in SAMINOR 1.

It is a limitation of our study that not all men and women who were invited to SAMINOR 1 attended and, further, that not all of those who attended SAMINOR 1 also participated in SAMINOR 2. In Norway, subjects who do not attend health surveys have been found to have lower socioeconomic status, higher prevalence of disability pension and higher mortality rates than attenders [53–55]. Table 1 demonstrates that the group of participants who attended both SAMINOR 1 and SAMINOR 2 differ in many ways from the group of men and women who attended only SAMINOR 1. They were somewhat older and a larger proportion were women, were married, had a higher income, reported better health generally and had lower self-reported prevalence of a history of myocardial infarction and angina pectoris. Furthermore, the smoking prevalence was lower and they were more physically active in their leisure time. This is in agreement with the findings of the Tromsø 2 survey, which found that young, unmarried men were over-represented among non-attenders [56].

There was a tendency towards lower body weight and waist circumference in participants attending both surveys than in men and women who took part in SAMINOR 1 only, but only minor age-adjusted differences and few statistically significant sex-and age-specific differences in weight and waist circumference between the participants who took part in both SAMINOR 1 and SAMINOR 2 (56% of the men and women who took part in SAMINOR 1) and those who only participated in SAMINOR 1 (44%). Based on this, we consider the participants included in the longitudinal analyses to be reasonably representative of the participants who took part in SAMINOR 1 with regard to the weight and waist circumference at baseline. However, particularly in the older age groups, some of the obese individuals may have died or been too ill to attend SAMINOR 2, which might have had some impact on our results [57]. On the other hand, the relationship between overweight and obesity (including abdominal obesity) with mortality is relatively weak in elderly people and underweight is associated with increased mortality as well [58].

We found a statistically significant increase in waist circumference in all age groups, but the oldest participants included in the longitudinal analyses were 69 years old at start of follow-up. Thus, we were not able to investigate how weight and waist circumference change with age in older individuals. It would have been interesting to have included older men and women in the analyses as recent results from the English Longitudinal Study of Ageing demonstrated that in old participants (aged 80 and above), the waist circumference did not increase or was even reduced with advancing age [59].

Furthermore, the study design makes it impossible to separate age effects from birth cohort effects and it is possible that the age effects we have presented are due to effects of when the subjects were born. However, as discussed in more detail above, the inverse relationship between age at baseline and longitudinal change in weight and waist circumference is in accordance with findings from previous studies.

## Conclusions

This longitudinal study over a 10-year period in rural Northern Norway finds that age at baseline, in the SAMINOR 1 Survey, is significantly and inversely associated with change in weight and waist circumference, irrespective of sex and Sami/non-Sami ethnicity. Emphasis should be put on prevention of obesity, particularly in younger people, as permanent weight loss is difficult in most obese people [60].

## Supporting information

S1 Table Mean (standard deviation, SD) body mass index (kg/m2) in SAMINOR 1 (2003–2004) and longitudinal changes in body mass index (kg/m2) (95% confidence interval) from SAMINOR 1 to SAMINOR 2 (2012–2014) in men and women born between 1934 and 1967 (aged 36–69 in SAMINOR 1) who attended both surveys. The SAMINOR Study (n = 3496).
(PDF)

S2 Table Mean (standard deviation, SD) body weight (kg) in SAMINOR 1 (2003–2004) and longitudinal changes in body weight (kg) (95% confidence interval) from SAMINOR 1 to SAMINOR 2 (2012–2014) according to ethnic group in men born between 1934 and 1967 (aged 36–69 in SAMINOR 1) who attended both surveys. The SAMINOR Study (n = 1538).
(PDF)

S3 Table Mean (standard deviation, SD) body weight (kg) in SAMINOR 1 (2003–2004) and longitudinal changes in body weight (kg) (95% confidence interval) from SAMINOR 1 to SAMINOR 2 (2012–2014) according to ethnic group in womena born between 1934 and 1967 (aged 36–69 in SAMINOR 1) who attended both surveys. The SAMINOR Study (n = 1955).
(PDF)

S4 Table Mean (standard deviation, SD) waist circumference (cm) in SAMINOR 1 (2003–2004) and longitudinal changes in waist circumference (cm) (95% confidence interval) from SAMINOR 1 to SAMINOR 2 (2012–2014) according to ethnic group in men born between 1934 and 1967 (aged 36–69 in SAMINOR 1) who attended both surveys. The SAMINOR Study (n = 1538).
(PDF)

S5 Table Mean (standard deviation, SD) waist circumference (cm) in SAMINOR 1 (2003–2004) and longitudinal changes in waist circumference (cm) (95% confidence interval) from SAMINOR 1 to SAMINOR 2 (2012–2014) according to ethnic group in womena born between 1934 and 1967 (aged 36–69 in SAMINOR 1) who attended both surveys. The SAMINOR Study (n = 1955).
(PDF)
