# Supplementary material for: A descriptive study of ten-year longitudinal changes in weight and waist circumference in the multi-ethnic rural Northern Norway. The SAMINOR Study, 2003-2014
Source: PLoS One. 2020 Feb 19;15(2):e0229234. doi: 10.1371/journal.pone.0229234 (PMC7029861; doi:10.1371/journal.pone.0229234)
Supplement: S1 Table — The SAMINOR Study (n = 3496). (PDF) [file pone.0229234.s001.pdf]

S1 Table. Mean (standard deviation, SD) body mass index (kg/m^2) in SAMINOR 1 (2003-2004) and longitudinal changes in body mass index (kg/m^2) (95 % confidence interval) from SAMINOR 1 to SAMINOR 2 (2012-2014) in men and women born between 1934 and 1967 (aged 36 – 69 in SAMINOR 1) who attended both surveys. The SAMINOR Study (n=3496).

	Birth year	Age in 2003 (years)	Number of participants	Body mass index, kg/m^2 (SD) in SAMINOR 1	Change in body mass index, kg/m^2 (95 % CI) between SAMINOR 1 and SAMINOR 2
Men					
	1964-1967	36-39	124	27.4 (3.5)	1.0 (0.6, 1.3)
	1959-1963	40-44	183	27.5 (3.5)	0.8 (0.5, 1.1)
	1954-1958	45-49	236	27.5 (3.5)	0.6 (0.3, 0.8)
	1949-1953	50-54	321	27.6 (3.9)	0.5 (0.3, 0.7)
	1944-1948	55-59	308	27.9 (3.9)	0.2 (0, 0.4)
	1939-1943	60-64	222	27.8 (3.6)	- 0.2 (- 0.4, 0.1)
	1934-1938	65-69	144	27.8 (3.4)	- 0.6 (- 0.9, - 0.3)
	All men	36-69	1538	27.7 (3.7)	0.3 (0.2, 0.4)
	p-value for linear trend			0.14	< 0.001
Women					
	1964-1967	36-39	163	26.1 (4.4)	1.1 (0.8, 1.5)
	1959-1963	40-44	270	26.9 (4.4)	0.8 (0.5, 1.0)
	1954-1958	45-49	342	27.3 (4.6)	0.5 (0.2, 0.8)
	1949-1953	50-54	399	27.4 (4.4)	0.1 (- 0.1, 0.4)
	1944-1948	55-59	364	28.4 (5.0)	0.1 (- 0.2, 0.3)
	1939-1943	60-64	241	28.6 (4.9)	-0.3 (- 0.6, 0)
	1934-1938	65-69	180	29.0 (4.6)	-0.9 (- 1.3, - 0.6)
	All women	36-69	1958	27.7 (4.7)	0.2 (0.1, 0.3)
	p-value for linear trend			< 0.001	< 0.001
