# Supplementary material for: A descriptive study of ten-year longitudinal changes in weight and waist circumference in the multi-ethnic rural Northern Norway. The SAMINOR Study, 2003-2014
Source: PLoS One. 2020 Feb 19;15(2):e0229234. doi: 10.1371/journal.pone.0229234 (PMC7029861; doi:10.1371/journal.pone.0229234)
Supplement: S2 Table — The SAMINOR Study (n = 1538). (PDF) [file pone.0229234.s002.pdf]

S2 Table. Mean (standard deviation, SD) body weight (kg) in SAMINOR 1 (2003-2004) and longitudinal changes in body weight (kg) (95 % confidence interval) from SAMINOR 1 to SAMINOR 2 (2012-2014) according to ethnic group in men born between 1934 and 1967 (aged 36 – 69 in SAMINOR 1) who attended both surveys. The SAMINOR Study (n=1538).

	Birth year	Age in 2003 (years)	Number of participants	Mean body weight, kg (SD) in SAMINOR 1	Change in weight, kg (95 % CI) between SAMINOR 1 and SAMINOR 2
Sami					
	1964-1967	36-39	50	82.9 (13.0)	3.1 (1.4, 4.9)
	1959-1963	40-44	91	81.3 (11.7)	2.1 (0.9, 3.2)
	1954-1958	45-49	100	80.2 (12.3)	1.4 (1.0, 2.4)
	1949-1953	50-54	130	81.0 (13.1)	0.6 (- 0.3, 1.5)
	1944-1948	55-59	127	80.1 (13.1)	- 0.6 (- 1.7, 0.6)
	1939-1943	60-64	74	80.1 (13.0)	- 0.9 (- 2.0, 0.2)
	1934-1938	65-69	59	78.3 (10.0)	- 3.0 (- 4.2, - 1.7)
	All Sami	36-69	631	80.5 (12.5)	0.4 (- 0.1, 0.8)
	p-value for linear trend			0.07	< 0.001
Non-Sami					
	1964-1967	36-39	74	86.1 (12.8)	3.2 (1.8, 4.6)
	1959-1963	40-44	92	85.5 (12.8)	3.2 (1.9, 4.6)
	1954-1958	45-49	136	85.3 (11.7)	1.9 (1.0, 2.9)
	1949-1953	50-54	191	85.0 (12.9)	1.4 (0.6, 2.2)
	1944-1948	55-59	181	84.7 (12.0)	0.9 (0, 1.7)
	1939-1943	60-64	148	82.8 (12.3)	- 0.8 (- 1.8, 0.1)
	1934-1938	65-69	85	81.0 (12.5)	- 1.8 (- 3.0, - 0.6)
	All non-Sami	36-69	907	84.4 (12.4)	1.0 (0.7, 1.4)
	p-value for linear trend			0.002	< 0.001
