# Supplementary material for: A descriptive study of ten-year longitudinal changes in weight and waist circumference in the multi-ethnic rural Northern Norway. The SAMINOR Study, 2003-2014
Source: PLoS One. 2020 Feb 19;15(2):e0229234. doi: 10.1371/journal.pone.0229234 (PMC7029861; doi:10.1371/journal.pone.0229234)
Supplement: S3 Table — The SAMINOR Study (n = 1955). (PDF) [file pone.0229234.s003.pdf]

S3 Table. Mean (standard deviation, SD) body weight (kg) in SAMINOR 1 (2003-2004) and longitudinal changes in body weight (kg) (95 % confidence interval) from SAMINOR 1 to SAMINOR 2 (2012-2014) according to ethnic group in women[a] born between 1934 and 1967 (aged 36 – 69 in SAMINOR 1) who attended both surveys. The SAMINOR Study (n=1955).

	Birth year	Age in 2003 (years)	Number of participants	Mean body weight, kg (SD) in SAMINOR 1	Change in weight, kg (95 % CI) between SAMINOR 1 and SAMINOR 2
Sami					
	1964-1967	36-39	67	67.0 (12.0)	3.5 (2.3,4.8)
	1959-1963	40-44	116	68.0 (11.3)	2.6 (1.6, 3.5)
	1954-1958	45-49	159	69.0 (11.1)	1.6 (0.7, 2.6)
	1949-1953	50-54	165	69.1 (12.0)	-0.3 (-1.1, 0.5)
	1944-1948	55-59	135	70.8 (11.4)	-1.5 (-2.5,- 0.4)
	1939-1943	60-64	78	71.0 (12.6)	-1.8 (- 3.0,- 0.6)
	1934-1938	65-69	69	70.5 (12.9)	-3.5 (- 4.6,- 2.4)
	All Sami	36-69	789	69.3 (11.8)	0.2 (- 0.2, 0.6)
	p-value for linear trend			0.007	< 0.001
Non-Sami					
	1964-1967	36-39	95	68.7 (11.7)	3.0 (1.8, 4.2)
	1959-1963	40-44	154	70.6 (12.0)	1.7 (0.6, 2.7)
	1954-1958	45-49	182	72.4 (12.8)	0.6 (- 0.4, 1.5)
	1949-1953	50-54	234	71.0 (11.6)	0.5 (- 0.3, 1.2)
	1944-1948	55-59	228	72.5 (13.7)	0.3 (- 0.6, 1.2)
	1939-1943	60-64	163	72.7 (12.8)	-1.0 (- 2.0, 0)
	1934-1938	65-69	110	71.8 (10.7)	-2.9 (- 4.3,- 1.6)
	All non-Sami	36-69	1166	71.6 (12.4)	0.3 (- 0.1, 0.7)
	p-value for linear trend			0.03	< 0.001

[a] Information about ethnic group was missing for 3 women.
