# Supplementary material for: A descriptive study of ten-year longitudinal changes in weight and waist circumference in the multi-ethnic rural Northern Norway. The SAMINOR Study, 2003-2014
Source: PLoS One. 2020 Feb 19;15(2):e0229234. doi: 10.1371/journal.pone.0229234 (PMC7029861; doi:10.1371/journal.pone.0229234)
Supplement: S4 Table — The SAMINOR Study (n = 1538). (PDF) [file pone.0229234.s004.pdf]

S4 Table. Mean (standard deviation, SD) waist circumference (cm) in SAMINOR 1 (2003-2004) and longitudinal changes in waist circumference (cm) (95 % confidence interval) from SAMINOR 1 to SAMINOR 2 (2012-2014) according to ethnic group in men born between 1934 and 1967 (aged 36 – 69 in SAMINOR 1) who attended both surveys. The SAMINOR Study (n=1538).

	Birth year	Age in 2003 (years)	Number of participants	Mean waist circumference, cm (SD) in SAMINOR 1	Change in waist circumference, cm (95 % CI), SAMINOR 1 to SAMINOR 2
Sami					
	1964-1967	36-39	50	90.4 (9.8)	7.5 (5.7, 9.4)
	1959-1963	40-44	91	90.3 (8.3)	7.1 (5.8, 8.5)
	1954-1958	45-49	100	90.9 (10.1)	6.2 (4.9, 7.5)
	1949-1953	50-54	130	91.3 (10.1)	6.5 (5.4, 7.6)
	1944-1948	55-59	127	93.8 (11.1)	5.1 (3.8, 6.5)
	1939-1943	60-64	74	94.0 (10.9)	6.0 (4.7, 7.3)
	1934-1938	65-69	59	94.1 (8.3)	3.8 (2.1, 5.5)
	All Sami	36-69	631	92.1 (10.0)	6.0 (5.5, 6.6)
	p-value for linear trend			< 0.001	0.001
Non-Sami					
	1964-1967	36-39	74	92.1 (9.3)	7.3 (5.8, 8.8)
	1959-1963	40-44	92	92.7 (9.9)	7.3 (5.8, 8.7)
	1954-1958	45-49	136	92.4 (8.1)	7.2 (6.1, 8.2)
	1949-1953	50-54	191	93.6 (10.1)	6.7 (5.8, 7.6)
	1944-1948	55-59	181	94.3 (9.7)	6.1 (5.3, 6.9)
	1939-1943	60-64	148	93.6 (8.7)	5.2 (4.3, 6.2)
	1934-1938	65-69	85	92.9 (10.3)	6.4 (5.0, 7.8)
	All non-Sami	36-69	907	93.3 (9.5)	6.5 (6.1, 6.9)
	p-value for linear trend			0.2	0.006
