# Supplementary material for: A descriptive study of ten-year longitudinal changes in weight and waist circumference in the multi-ethnic rural Northern Norway. The SAMINOR Study, 2003-2014
Source: PLoS One. 2020 Feb 19;15(2):e0229234. doi: 10.1371/journal.pone.0229234 (PMC7029861; doi:10.1371/journal.pone.0229234)
Supplement: S5 Table — The SAMINOR Study (n = 1955). (PDF) [file pone.0229234.s005.pdf]

S5 Table. Mean (standard deviation, SD) waist circumference (cm) in SAMINOR 1 (2003-2004) and longitudinal changes in waist circumference (cm) (95 % confidence interval) from SAMINOR 1 to SAMINOR 2 (2012-2014) according to ethnic group in women[a] born between 1934 and 1967 (aged 36 – 69 in SAMINOR 1) who attended both surveys. The SAMINOR Study (n=1955)

	Birth year	Age in 2003 (years)	Number of participants	Mean waist circumference, cm (SD) in SAMINOR 1	Change in waist circumference, cm (95 % CI), SAMINOR 1 to SAMINOR 2
Sami					
	1964-1967	36-39	67	81.5 (11.6)	10.4 (8.8,12.1)
	1959-1963	40-44	116	82.0 (10.1)	10.5 (9.2,11.7)
	1954-1958	45-49	159	83.9 (11.9)	10.0 (8.9,11.2)
	1949-1953	50-54	165	85.1 (11.2)	7.8 (6.8, 8.8)
	1944-1948	55-59	135	87.5 (11.0)	7.0 (5.6, 8.4)
	1939-1943	60-64	78	89.1 (11.5)	6.5 (4.8, 8.3)
	1934-1938	65-69	69	90.0 (10.7)	4.6 (3.1, 6.1)
	All Sami	36-69	789	85.3 (11.5)	8.3 (7.8, 8.8)
	p-value for linear trend			< 0.001	< 0.001
Non-Sami					
	1964-1967	36-39	95	81.0 (10.7)	9.7 (8.2, 11.2)
	1959-1963	40-44	154	80.8 (10.3)	9.7 (8.5, 11.0)
	1954-1958	45-49	182	83.1 (10.7)	9.4 (8.2, 10.5)
	1949-1953	50-54	234	83.8 (10.5)	8.6 (7.7, 9.5)
	1944-1948	55-59	228	86.1 (13.0)	7.9 (6.8, 9.0)
	1939-1943	60-64	163	86.5 (11.5)	8.4 (7.1, 9.6)
	1934-1938	65-69	110	87.3 (10.9)	5.8 (4.3, 7.2)
	All non-Sami	36-69	1166	84.2 (11.4)	8.5 (8.1, 9.0)
	p-value for linear trend			< 0.001	< 0.001

[a] Information about ethnic group was missing for 3 women.
